# 

**Published:** 1893-06

**Authors:** 


### CONTENTS.

ORIGINAL ARTICLES—Mechanical Treatment of some Skin Anomalies, by M. B. Hutchins, M. D., Atlanta, Ga .......... 285
Report of a Case of Traumatic Empyena, by N. T. Dulaney, M.D., Bristol, Tenn .......... 293
Case of Impending Diabetic Coma, by H. McHatton, M. D., Macon, Ga. .......... 296
Syphilis from a Sociological Standpoint, by W. F. Westmoreland, M. D., Atlanta, Ga .......... 299
CORRESPONDENCE—New York Letter. .......... 303
SOCIETY REPORTS—Clinical Society of Maryland .......... 308
Philadelphia Academy of Surgery .......... 314
BOOK REVIEWS—Diet for the Sick, by Miss E. Hibbard and Mrs. Emma Drant. .......... 321
Syphilis and the Nervous System, by W. R. Gower, M. D .......... 321
A Text-Book of the Theory and Practice of Medicine, Edited by William Pepper, M. D .......... 322
A Hand-Book of Diseases of the Eye and their Treatment, H. R. Swanzy, A. M., M. D. .......... 323
A Practical Treatise on Materia Medica and Therapeutics, by John V. Shoemaker, A. M., M. D .......... 324
SELECTIONS AND ABSTRACTS—Protection of Children in France .......... 307
Treatment of Bubo by Excision .......... 325–326
EDITORIALS—Scarlet Fever Scare .......... 327
HIGHER MEDICAL EDUCATION .......... 328
WOMAN .......... 329
PRESCRIPTIONS. .......... 334–335
SPECIAL NOTES .......... 336–338

